# Exploring Genetic Interactions in Colombian Women with Polycystic Ovarian Syndrome: A Study on SNP-SNP Associations

**DOI:** 10.3390/ijms25179212

**Published:** 2024-08-25

**Authors:** Maria Camila Alarcón-Granados, Gloria Eugenia Camargo-Villalba, Maribel Forero-Castro

**Affiliations:** 1 Faculty of Sciences, Universidad Pedagógica y Tecnológica de Colombia, Tunja 150003, Colombia; mcamila.alarcon02@gmail.com; 2 Medicine Program, Faculty of Health Sciences, Universidad de Boyacá, Tunja 150003, Colombia; gloriacamargo@uniboyaca.edu.co

**Keywords:** epistasis, PCOS, polymorphisms, multifactor dimensionality reduction

## Abstract

Polycystic ovary syndrome (PCOS) is an endocrine and metabolic disorder with high prevalence in women around the world. The identification of single-nucleotide polymorphisms (SNPs) through genome-wide association studies has classified it as a polygenic disease. Most studies have independently evaluated the contribution of each SNP to the risk of PCOS. Few studies have assessed the effect of epistasis among the identified SNPs. Therefore, this exploratory study aimed to evaluate the interaction of 27 SNPs identified as risk candidates and their contribution to the pathogenesis of PCOS. The study population included 49 control women and 49 women with PCOS with a normal BMI. Genotyping was carried out through the MassARRAY iPLEX single-nucleotide polymorphism typing platform. Using the multifactor dimensionality reduction (MDR) method, the interaction between SNPs was evaluated. The analysis showed that the best interaction model (*p* < 0.0001) was composed of three loci (rs11692782-*FSHR*, rs2268361-*FSHR*, and rs4784165-*TOX3*). Furthermore, a tendency towards synergy was evident between rs2268361 and the SNPs rs7371084–rs11692782–rs4784165, as well as a redundancy in rs7371084–rs11692782–rs4784165. This pilot study suggests that epistasis may influence PCOS pathophysiology. Large-scale analysis is needed to deepen our understanding of its impact on this complex syndrome affecting thousands of women.

## 1. Introduction

Polycystic ovary syndrome (PCOS) is a complex chronic disorder that manifests in women of reproductive age. The prevalence of PCOS varies according to the diagnostic criteria used and the study population. Globally, the prevalence ranges from 5 to 15% [1]. The Rotterdam 2003 criteria have been the most used for the diagnosis of PCOS. These include the presence of at least two of three characteristics: clinical/biochemical hyperandrogenism, polycystic ovarian morphology, and oligo/amenorrhea [2].

The heterogeneity of PCOS is manifested throughout a woman’s life through reproductive, metabolic, dermatological, and psychological consequences [3]. Although research efforts for this disorder are considerable, the etiology remains unknown [4]. It has been identified that environmental and genetic factors contribute to the progression of the disease. From a genetic perspective, genome-wide association studies (GWASs) have been established as the most effective approach to identify single-nucleotide polymorphisms (SNPs) in complex diseases like PCOS [5]. However, the identified SNPs generally show modest effects on the disease risk, which is known as a “missing heritability” problem. In response to this challenge, identifying SNP-SNP interactions (also called epistasis) has been proposed, as complex diseases are determined by multiple genetic factors that interact with each other [6].

Various methods have been described for the analysis of these interactions, mostly based on statistical regression models [7]. However, these models require a priori genetic models and face challenges with data dimensionality, given that an increase in the number of variables (SNPs) exponentially increases higher-order interactions [8]. Increasing the sample size reduces this problem and allows for a robust estimation of interactions. However, this results in additional high costs [7].

The multifactor dimensionality reduction (MDR) method developed by Ritchie et al. [9] was the first machine learning approach proposed as an alternative to small sample sizes and limitations of statistical methods in gene–gene interaction analyses. MDR is a nonparametric method used in case–control studies, which reduces the dimensionality of SNP genotypes by grouping them into high- and low-risk groups. This diminishes type I and II errors [10]. Additionally, MDR can detect high-order interactions, even in the absence of statistically significant main effects [11]. Since MDR does not assume a specific inheritance model, it selects the best SNP model among all possible combinations through a cross-validation procedure, achieving maximum balanced accuracy. Permutation tests allow one to identify whether the model is statistically significant [8,12].

For PCOS, there are large amounts of genomic data and studies focusing on detecting SNPs in isolation. However, few studies have explored interactions between polymorphisms for this complex disorder. Therefore, this research aimed to evaluate the epistatic effect of 27 SNPs from the genes *THADA*, *LHCGR*, *FSHR*, *DENND1A*, *YAP1*, *HMGA2*, *ERBB3*, *AMHR2*, *TOX3*, *INSR*, and *AMH* in a sample of Colombian women with PCOS.

## 2. Results

### 2.1. Characteristics of the Study Sample

Table 1 and Table 2 present the clinical, endocrine, and metabolic characteristics of the study sample, previously reported by Alarcón-Granados et al. [13]. Significant differences were observed in weight, with the PCOS group having a higher median weight (60.8 kg) compared to the controls (60 kg) (*p* = 0.037). No significant differences were noted in height and body mass index (BMI) between the groups. It should be noted that the PCOS group had an average BMI within the normal range (23.16 kg/m^2^), representing the lean PCOS phenotype. This subgroup is not reflective of the broader PCOS population, which generally includes more women with overweight or obesity.

The PCOS group had significantly higher levels of follicle-stimulating hormone (FSH), antimüllerian hormone (AMH), luteinizing hormone (LH), estradiol (E_2_), total ovarian volume, and total antral follicular count (AFC) compared to controls (all *p* < 0.0001). Family history data revealed higher incidences of polycystic ovaries and endometriosis among the PCOS group. Reproductive features indicated significantly fewer pregnancies and higher incidences of early pregnancy loss in women with PCOS compared to controls (Table 1).

In women with PCOS, hyperandrogenism was evident from elevated levels of androstenedione (1.49 ± 0.59 ng/mL), DHEAS (152.8 ± 64.51 µg/dL), and free testosterone (median 1.34 pg/mL), as shown in Table 2, with clinical manifestations including acne (60%), facial hair (68%), and abdominal hair (60%). Amenorrhea, or the absence of menstrual periods, is reflected in the significantly longer menstrual cycle length in women with PCOS (31 days) compared to controls (28 days, *p* < 0.0001), with 60% experiencing menstrual bleeding cessation for more than 3 months and 50% reporting multiple menstrual bleeds in one month. Ovarian ultrasound findings, crucial for PCOS diagnosis, show a significantly higher total ovarian volume (12.25 cm^3^ vs. 7.61 cm^3^, *p* < 0.0001) and total antral follicular count (median 27 vs. 16, *p* < 0.0001), indicating the typical polycystic ovarian morphology associated with the syndrome.

### 2.2. Epistasis Analysis

The basic information related to the 27 SNPs included in this study is shown in Table 3. The correlation between polymorphisms in the *THADA*, *LHCGR*, *FSHR*, *DENND1A*, *YAP1*, *HMGA2*, *ERBB3*, *AMHR2*, *TOX3*, *INSR*, and *AMH* genes and the risk to PCOS in the allele model was evaluated. However, no statistically significant difference was observed between polymorphisms and PCOS risk (*p* > 0.05).

**Figure 1:**
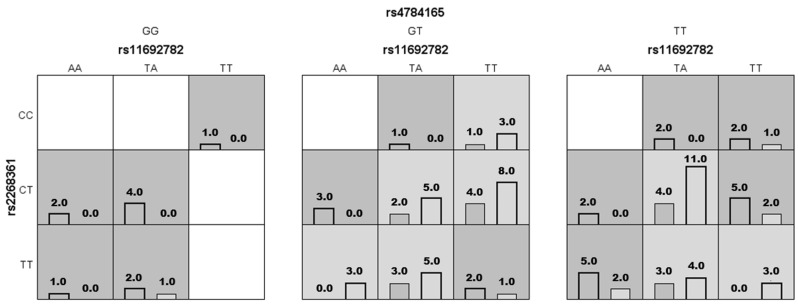
Distribution of high-risk and low-risk genotypes in the best three-*locus* model. Each cell shows counts of the control group on the left and the PCOS group on the right. Dark-gray cells represent high risk and light cells represent low risk.

Table 4 summarizes the results of the SNP-SNP interaction analysis. We found that the three-locus model including rs11692782-*FSHR*, rs2268361-*FSHR*, and rs4784165-*TOX3* was the best model with a cross-validation consistency = 7/10, testing balanced accuracy = 0.6327, and *p* < 0.0001. Figure 1 details the combinations of genotypes associated with PCOS risk in this model. We identified interactions in high-risk genotypes for rs4784165, rs11692782, and rs2268361 such as (GG + TT + CC), (GG + AA + CT), (GG + TA + CT), (GG + AA + TT), (GG + TA + TT), (GT + TA + CC), (GT + AA + CT), (GT + TT + TT + TT), (TT + TA + CC), (TT + TT + CC), (TT + AA + CT), (TT + TT + TT + CT), and (TT + AA + TT), respectively, and low-risk genotypes such as (GG + AA + CC), (GG + TA + CC), (GG + TT + CT), (GG + TT + TT + TT), (GT + AA + CC), and (TT + AA + CC), respectively.

**Figure 2:**
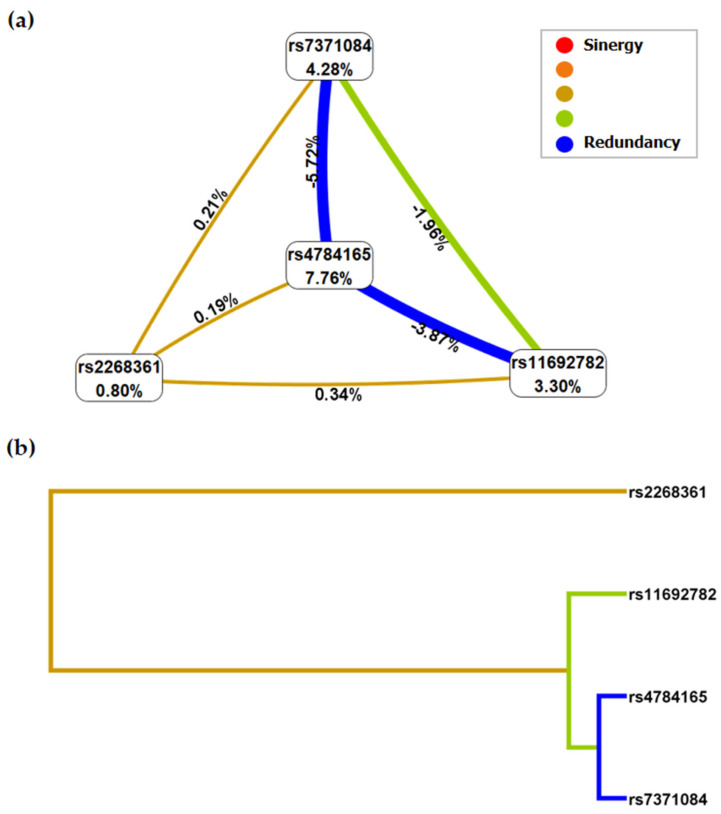
Entropy analysis. (**a**) Interaction map for PCOS risk. (**b**) Dendrogram between SNPs in PCOS patients. The coloring scheme represents a continuum from synergy (red, representing a non-additive interaction) to redundancy (blue, representing loss of information).

The entropy analysis that evaluates what types of effects are represented in the model is detailed in Figure 2. In this analysis, a new SNP was included in the model (rs7371084-*LHCGR*). In the interaction map (Figure 2a), each node represents an SNP, as well as the individual entropy percentage for each polymorphism (main effects). The values between the nodes represent the interaction effects between SNPs. Positive entropy values (represented by red or orange lines) between polymorphisms indicate information gain or synergy, and negative values (represented by yellow or green lines) indicate redundancy or independence. Our results show a synergistic interaction between rs2268361 and rs7371084 (0.21%), rs4784165 (0.19%), and rs11692782 (3.30%). These last three SNPs present a redundancy effect among themselves. The dendrogram representing the interactions between SNPs (Figure 2b) groups the polymorphisms with the highest redundancy (rs4784165–rs7371084) and synergy (rs2268361).

## 3. Discussion

This is the first study in a sample of Colombian women that applies the MDR method to identify SNP-SNP interactions in 27 variants located in the genes *THADA*, *LHCGR*, *FSHR*, *DENND1A*, *YAP1*, *HMGA2*, *ERBB3*, *AMHR2*, *TOX3*, *INSR*, and *AMH*, associated with PCOS. In the individual risk analysis under the allelic model, no statistically significant differences were identified between women with PCOS and controls. In previous published [13,14] and unpublished pilot studies for the same cohort, we identified a negative association between rs7371084-*LHCGR* and rs4784165-*TOX3*, and a positive association between the SNPs rs10986105, rs10818854, rs7857605, and rs12337273 in the *DENND1A* gene and the risk of PCOS.

Using the MDR method, it was evident that the three-locus model including rs11692782-*FSHR*, rs2268361-*FSHR*, and rs4784165-*TOX3* was the best (*p* < 0.001). This model presented an OR (95% CI) of 11.29 (4.183–30.49), a value that indicates a significant increase in the pathogenesis of the syndrome. These results were confirmed when creating the interaction map and dendrogram, where the SNP rs7371084-*LHCGR* was included. A tendency towards synergy was observed between rs2268361 and the SNPs rs7371084–rs11692782–rs4784165. This suggests that their combined effects are larger or different than would be expected by simply adding the individual effects of each variant [15]. The synergy not only suggests a greater combined effect between SNPs but may also indicate a functional relationship between the variants and a higher penetrance of the PCOS phenotype [16].

The genes involved in this interaction were *FSHR*, *LHCGR*, and *TOX3*. The relationship between *FSHR* and *LHCGR* has been well described since both encode receptors for key hormones in the regulation of the menstrual cycle and reproductive function: follicle-stimulating hormone (FSH) and luteinizing hormone (LH). In healthy women, the LH:FSH ratio is 1 [17]. During the menstrual cycle, FSH and LH act together to regulate the growth and maturation of ovarian follicles, as well as ovulation [18]. FSH stimulates the growth of follicles in the ovaries, while LH induces ovulation and helps maintain the corpus luteum, which produces progesterone [19]. The expression and activity of these receptors are coordinated and regulated in a complex manner to guarantee adequate maturation of the follicles, ovulation, and preparation of the endometrium for implantation of the fertilized egg [20].

In women with PCOS, one of the most common hormonal alterations is increased levels of LH. This increase causes a high LH:FSH ratio, from 1 to 5.5 [21]. Elevated LH stimulates the production of androgens in the theca cells of the ovary, which can contribute to hyperandrogenemia, manifesting in symptoms such as hirsutism and acne [22]. Meanwhile, FSH generally remains normal or low compared to LH. In women with PCOS, the altered LH:FSH ratio prevents adequate maturation of the follicles, leading to anovulatory cycles [23]. This hormonal dysregulation contributes to menstrual irregularity, a cardinal symptom of PCOS [24], and reproductive problems such as infertility or difficulties conceiving, which are common concerns among women with this condition [25].

On the other hand, *TOX3* is a transcription factor that plays a role in regulating gene expression in various cells and tissues, including reproductive ones [26]. Variants in *TOX3* have been associated with an increased risk in women with PCOS, and their possible involvement in the regulation of ovarian function and fertility has been suggested [27]. However, at the functional level, the interaction of *TOX3* with *FSHR* and *LHCGR* has not been described.

In contrast, it was observed that the interaction between rs7371084–rs11692782–rs4784165 was represented by redundancy values, which means that the combined effects of the SNPs are similar to the sum of the individual effects of each SNP [28]. Redundancy may be at a functional level, suggesting that these variants affect similar or overlapping biological pathways or processes in the development of PCOS [29], or indicate the presence of compensatory mechanisms, where a variant that increases the risk of PCOS could be compensated due to the presence of another variant that has a protective or neutralizing effect [30]. Similar results were reported by Thathapudi et al. [31] who established a weak interaction between *LHCGR* and *FSHR/CAPN-10* variants in women with PCOS.

Considering that PCOS is a multifactorial and complex disorder, presenting a wide variability in symptoms and severity, studies on epistasis are essential for understanding the genetic basis and phenotypic variability of the disease. These studies can identify new therapeutic targets and improve individual risk prediction, which could have significant implications for developing more effective prevention and treatment strategies for PCOS.

While our study provides an initial exploration of epistatic effects, the small sample size and limited number of SNPs per gene constrain the depth of our analysis. Future research with larger and more diverse samples is needed to robustly assess these interactions and their implications. In particular, future studies with larger sample sizes could investigate the impact of gene interactions on phenotypic features of PCOS in both lean and obese cohorts of women. To increase the number of PCOS patients available for analysis, it would be beneficial to establish connections and networks with research groups, associations, and organizations focused on women’s sexual and reproductive health. Collaborative efforts and engagement with these networks could facilitate access to larger and more diverse patient populations.

Regarding the limitation of our study’s focus on Colombian women, we acknowledge that genetic variations and disease manifestations can differ across populations. This regional focus may impact the generalizability of our findings. Addressing this, we suggest that future studies should include diverse populations to better understand the global applicability of the results and to overcome these limitations. Furthermore, the insights gained from our study, despite its limitations, provide a foundation for exploring the clinical applications of epistatic interactions in PCOS. With broader and more inclusive research, there is potential for translating these findings into improved risk assessment and targeted treatment strategies.

## 4. Materials and Methods

### 4.1. Study Participants

We conducted an exploratory case–control study in Colombia with a sample of 49 control women and 49 women with a confirmed diagnosis of PCOS. The characteristics of the sample and the inclusion and exclusion criteria have been detailed previously [13].

### 4.2. SNP Selection and Genotyping

We included 27 SNPs from 11 genes widely reported as risk candidates for PCOS in different studies [32,33]. According to the manufacturer’s instructions, we used the Invisorb R Spin Universal Kit (Stratec Molecular, Berlin, Germany) to extract total genomic DNA from the peripheral blood of the participants. DNA concentration was measured using an EPOCHTM2 Microplate Spectrophotometer (Biotek, Winooski, VT, USA). Genotyping was performed using the MassARRAY iPLEX single-nucleotide polymorphism typing platform (Agena Bioscience, San Diego, CA, USA). This platform employs matrix-assisted laser desorption ionization time-of-flight mass spectrometry (MALDI-TOF MS) in conjunction with single-base extension polymerase chain reaction (PCR) to enable high-throughput multiplex detection of SNPs [34]. The design of extension primers was performed using the Assay Design Suite (ADS) software version 2.0, and the allelic discrimination, after the iPLEX reaction, was performed using the Typer software version 4.0. Design details are shown in Table 5.

### 4.3. Statistic and SNP-SNP Interaction Analysis

The R Studio software version 4.2.3 was used to evaluate the risk to PCOS of the polymorphisms under the allelic model. The minor allele frequency (MAF) for each SNP, Hardy–Weinberg Equilibrium (HWE), odds ratio (OR), and 95% confidence interval (95% CI) were determined. The *p*-value was estimated using a chi-square test.

SNP-SNP interactions in PCOS risk were evaluated between the 27 SNPs by the nonparametric model-free multifactor dimensionality reduction (MDR) method using the MDR software version 3.0.2 (open-source version available at https://www.epistasis.org, accessed on 5 February 2024).

The SNP combination with maximum cross-validation consistency (CVC) and test accuracy was considered the best model [35]. For the best model, a dendrogram and an interaction map for PCOS risk were created to represent the interactions between SNPs. In the interaction map, each node corresponds to a polymorphism and the individual effect value of each SNP. The values between SNPs represent the entropy and interaction strength. Negative values mean redundancy and positive values mean synergy between polymorphisms. Additionally, the OR, 95% CI, and *p*-value were calculated in the best models obtained. Significance was considered with a *p*-value < 0.05.

## 5. Conclusions

This exploratory study in a sample of Colombian women evaluated 27 polymorphisms previously identified as risk candidates for PCOS. Through the MDR method, we identified that the best interaction model was rs11692782-*FSHR*, rs2268361-*FSHR*, and rs4784165-*TOX3*. The interaction graphs showed a tendency towards synergy between rs2268361 and the SNPs rs7371084–rs11692782–rs4784165, and a tendency towards redundancy between rs7371084–rs11692782–rs4784165. The above demonstrates that polymorphisms in complex diseases can interact with each other and contribute to the pathogenesis of the disease. Therefore, it is necessary to carry out large-scale studies that allow us to elucidate, even at a functional level, the effect of epistasis in PCOS.

## Figures and Tables

**Table 1:** Comparative clinical and hormonal profile of PCOS and control groups. Adapted from Alarcón-Granados et al. [13].

	PCOS Group	Control Group	*p*-Value
Case number	49	49	
Age (years)	28 (24–33)	27 (24–30)	0.448 ^†^
Weight (kg)	60.8 (55–74)	60 (52–64)	0.037 ^†^
Height (m)	1.62 (1.59–1.66)	1.6 (1.56–1.64)	0.064 ^†^
BMI (kg/m^2^)	23.16 (21.48–25.6)	22.6 (20–24.98)	0.22 ^†^
Menarche (years)	13 (12–14)	12 (11.5–14)	0.185 ^†^
Menstrual cycle length (days)	31 (29.5–45)	28 (28–30)	<0.0001 ^†^
Period length (days)	5 (4–8)	5 (4–5)	0.129 ^†^
FSH (mUl/mL)	5.95 ± 3.47	9.5 ± 5	<0.0001 ^††^
AMH (ng/mL)	8.02 (5.07–12.55)	4.87 (3.05–6.77)	<0.0001 ^†^
LH (mUl/mL)	6.8 (4.55–10.3)	3.2 (2.12–5.17)	<0.0001 ^†^
LH/FSH ratio	1.27 (0.83–1.74)	0.38 (0.18–0.64)	<0.0001 ^†^
TSH (mUl/mL)	1.67 (1.29–2.69)	1.65 (1.05–2.47)	0.284 ^†^
E_2_ (pg/mL)	53.3 (32.72–72.87)	29.7 (15–40.6)	<0.0001 ^†^
Total ovarian volume (cm^3^)	12.25 (9.62–18.75)	7.61 (6.63–9.47)	<0.0001 ^†^
Total AFC (number of follicles)	27 (23–34,75)	16 (13–20)	<0.0001 ^†^
Family History			
Family history of polycystic ovaries	22 (44.8%)	6 (12.24%)	<0.0001 ^†††^
Family history of endometriosis	10 (20.4%)	4 (8.16%)	0.013 ^†††^
Family history of breast and ovarian cancer	10 (20.4%)	6 (12.24%)	0.196 ^†††^
Reproductive traits			
Pregnancies	12 (24.48%)	33 (67.34%)	<0.0001 ^†††^
Early pregnancy loss	8 (16.32%)	2 (4.08%)	0.045 ^†††^
Spontaneous abortion	7 (14.28%)	2 (4.08%)	0.091 ^†††^

Abbreviations: BMI: body mass index; FSH: follicle-stimulating hormone; AMH: antimüllerian hormone; LH: luteinizing hormone; TSH: thyroid-stimulating hormone; E_2_: estradiol; AFC: antral follicular count. ^†^ Mann-Whitney U-test (nonparametric variables). Data are expressed as median (interquartile range). ^††^ Student’s *t*-test (parametric variables). Data are expressed as mean ± standard deviation. ^†††^ Fisher’s exact test and chi-square test were used for analyzing the associations between categorical variables.

**Table 2:** PCOS endocrine–metabolic parameters and clinical symptoms. Adapted from Alarcón-Granados et al. [13].

**Endocrine–Metabolic Parameters**	**Value ^†^**
Androstenedione (ng/mL)	1.49 ± 0.59
DHEAS (μg/dL)	152.8 ± 64.51
Free testosterone (pg/mL)	1.34 (0.91–2.40)
Fasting insulin (μUl/mL)	4.68 (2.62–9.16)
Post-meal insulin (μUl/mL)	28.3 (13.1–43.6)
Fasting blood glucose (mg/dL)	83.91 ± 8.51
Post-meal glucose (mg/dL)	80.5 (72.5–95)
HOMA-IR	0.84 (0.48–1.95)
HOMA-IS	0.49 (0.02–0.08)
Glycosylated hemoglobin (%)	5.24 (5.01–5.74)
**Clinical Parameters**	**n (%) ^††^**
Acne	30 (60%)
Hair loss	43 (86%)
Facial hair	34 (68%)
Abdominal hair	30 (60%)
Fatty discharge from scalp and face	33 (66%)
Acanthosis nigricans	10 (20%)
Cystic lesion resection	2 (4%)
Menstrual bleeding stopped for more than 3 months	30 (60%)
Multiple menstrual bleeds in one month	25 (50%)
Postcoital bleeding	5 (10%)
Dysmenorrhea	29(58%)

Abbreviations: DHEAS: Dehydroepiandrosterone sulfate; HOMA-IR: Homeostasis model Assessment—Insulin resistance; HOMA-IS: Homeostasis model Assessment—Insulin sensitive. ^†^ The parametric variables are expressed as mean ± standard deviation, and nonparametric variables are expressed as median (interquartile range). ^††^ Data are expressed as a number of cases (percentage).

**Table 3:** Basic information of 27 polymorphisms included in this study.

Gene	SNP ID	Chr	Position	Consequence	Alleles	MAF		HWE-p	OR (95% CI)	*p*-Value
						Case	Control			
*THADA*	rs13429458	2	43411699	Intron Variant	A > C	0.12	0.1	0.34	1.23 (0.50–2.99)	0.65
*THADA*	rs12478601	2	43494369	Intron Variant	C > T	0.43	0.39	0.53	1.18 (0.67–2.09)	0.56
*THADA*	rs12468394	2	43334022	Intron Variant	C > A	0.32	0.32	0.49	1.01 (0.55–1.86)	0.97
*THADA*	rs6544661	2	43484786	Intron Variant	A > G	0.44	0.4	0.84	1.18 (0.67–2.09)	0.56
*THADA*	rs11891936	2	43305163	Intron Variant	C > T	0.12	0.13	0.65	0.91 (0.39–2.11)	0.83
*LHCGR*	rs13405728	2	48751020	Intron Variant	A > G	0.1	0.14	0.63	0.68 (0.2–1.6)	0.38
*LHCGR*	rs7371084	2	48712814	Intron Variant	T > C	0.13	0.21	0.29	0.56 (0.26–1.19)	0.13
*LHCGR*	rs4953616	2	48714289	Intron Variant	T > C	0.32	0.28	0.63	1.21 (0.6–2.25)	0.53
*LHCGR*	rs2293275	2	48694236	Missense Variant	C > T	0.36	0.29	0.49	1.52 (0.30–7.53)	0.28
*LHCGR*	rs6732721	2	48738464	Intron Variant	T > C	0.12	0.16	0.4	0.71 (0.32–1.60)	0.41
*FSHR*	rs2268361	2	48974473	Intron Variant	T > C	0.35	0.41	0.28	0.77 (0.43–1.37)	0.38
*FSHR*	rs2349415	2	49020693	Intron Variant	C > T	0.4	0.31	1	1.50 (0.83–2.70)	0.18
*FSHR*	rs11692782	2	49064754	Intron Variant	T > A	0.37	0.48	0.84	0.63 (0.36–1.12)	0.11
*DENND1A*	rs2479106	9	123762933	Intron Variant	A > G	0.22	0.23	0.77	0.94 (0.49–1.84)	0.87
*DENND1A*	rs10818854	9	123684499	Intron Variant	G > A	0.09	0.04	0.35	2.38 (0.71–7.99)	0.15
*DENND1A*	rs10986105	9	123787676	Intron Variant	T > G	0.09	0.03	0.3	3.20 (0.84–12.21)	0.07
*DENND1A*	rs12337273	9	123804666	Intron Variant	A > G	0.08	0.03	0.26	2.81 (0.72–10.94)	0.12
*DENND1A*	rs1778890	9	123769476	Intron Variant	T > C	0.15	0.14	1	1.08 (0.49–2.39)	0.84
*DENND1A*	rs1627536	9	123780425	Intron Variant	A > T	0.23	0.24	1	0.95 (0.49–1.82)	0.87
*DENND1A*	rs7857605	9	123745334	Intron Variant	T > C	0.09	0.04	0.35	2.38 (0.71–7.99)	0.15
*YAP1*	rs1894116	11	102199908	Intron Variant	A > G	0.03	0.05	1	0.59 (0.14–2.53)	0.47
*HMGA2*	rs2272046	12	65830681	Intron Variant	A > C	0.03	0.01	1	3.06 (0.31–29.97)	0.31
*ERBB3*	rs2292239	12	56088396	Intron Variant	G > T	0.21	0.22	1	0.94 (0.48–1.85)	0.86
*AMHR2*	rs2272002	12	53424132	Intron Variant	T > A	0.07	0.1	1	0.68 (0.25–1.86)	0.45
*TOX3*	rs4784165	16	52313907	Intron Variant	T > G	0.28	0.37	0.65	0.66 (0.36–1.20)	0.17
*INSR*	rs2059807	19	7166098	Intron Variant	G > A	0.45	0.5	1	0.82 (0.47–1.43)	0.47
*AMH*	rs10407022	19	2249478	Missense Variant	T > G	0.16	0.19	0.5	0.81 (0.39–1.69)	0.58

Chr: chromosome; MAF: minor allele frequency; HWE: Hardy–Weinberg Equilibrium.

**Table 4:** Best MDR models of SNP-SNP interactions.

Model	Bal. Acc. CV Training	Bal. Acc. CV Testing	CV Consistency	OR (95% CI)	*p*-Value
rs7371084	0.61	0.4184	5/10	2.59 (1.077–6.232)	0.0312
rs11692782, rs4784165	0.6667	0.4082	3/10	3.78 (1.6–8.949)	0.002
rs11692782, rs2268361, rs4784165	0.7574	0.6327	7/10	11.29 (4.183–30.49)	*p* < 0.0001

**Table 5:** Design details for SNP genotyping.

SNP_ID	2nd-PCRP	1st-PCRP	AMP_LEN	UP_CONF	MP_CONF	Tm (NN)	PcGC	UEP_DIR	UEP_MASS	UEP_SEQ	EXT1_CALL	EXT1_MASS	EXT1_SEQ	EXT2_CALL	EXT2_MASS	EXT2_SEQ
rs10407022	ACGTTGGATGTCTTCCGAGAAGACTTGGAC	ACGTTGGATGAGCTGCTGCCATTGCTGTC	110	95.6	72.3	53.7	66.7	F	4538.0	ACTGGCCTCCAGGCA	G	4825.2	ACTGGCCTCCAGGCAG	T	4865.1	ACTGGCCTCCAGGCAT
rs10818854	ACGTTGGATGGTGCTTAAAGGTGGGAATGC	ACGTTGGATGCACTGCCTTCTGTAAGACAC	90	99.6	72.3	47.5	60.0	R	4664.0	GGGAATGCTTGCTGG	G	4911.2	GGGAATGCTTGCTGGC	A	4991.1	GGGAATGCTTGCTGGT
rs2349415	ACGTTGGATGAAAAACAGGTGTCAGGCTGG	ACGTTGGATGACAACTCCACGATCTAGGAC	92	99.7	72.3	46.0	50.0	F	4952.2	GTCAGGCTGGATTTGA	C	5199.4	GTCAGGCTGGATTTGAC	T	5279.3	GTCAGGCTGGATTTGAT
rs2272002	ACGTTGGATGGTAAGGGTGAAGGATAGAGC	ACGTTGGATGTATGGTAAAGCCACAGGAGG	111	99.5	72.3	55.1	73.3	F	5162.4	ttTCCCCATGGCAGGGC	A	5433.6	ttTCCCCATGGCAGGGCA	T	5489.5	ttTCCCCATGGCAGGGCT
rs12468394	ACGTTGGATGTCTGTGGCTAACTGCAGAAG	ACGTTGGATGAATGCTGTTTTCAGCTGTTG	88	93.8	72.3	45.9	50.0	R	5241.4	gCTGCAGAAGTTCTGGT	C	5528.6	gCTGCAGAAGTTCTGGTG	A	5568.5	gCTGCAGAAGTTCTGGTT
rs1627536	ACGTTGGATGCATGGCAATAGTAAGTGCTC	ACGTTGGATGCATCCAGTGAATGATGGTGC	116	97.6	72.3	45.9	44.4	R	5360.5	CTTCCCTTCTTAATCCGA	T	5631.7	CTTCCCTTCTTAATCCGAA	A	5687.6	CTTCCCTTCTTAATCCGAT
rs13405728	ACGTTGGATGCTTCAATATCCTGGGCTTAC	ACGTTGGATGGATTTAGAAACCTGCTCTGG	120	95.6	72.3	49.2	42.1	R	5762.8	CCATAATGCAGCCATTTGT	G	6010.0	CCATAATGCAGCCATTTGTC	A	6089.9	CCATAATGCAGCCATTTGTT
rs6544661	ACGTTGGATGAACACATATAGGTGCTCCTC	ACGTTGGATGTCCTCTCATTAGAACATCTC	93	92.8	72.3	45.6	52.9	F	5770.7	gcGGTGCTCCTCTTAGTAC	A	6042.0	gcGGTGCTCCTCTTAGTACA	G	6058.0	gcGGTGCTCCTCTTAGTACG
rs2293275	ACGTTGGATGCAATGTGAAAGCACAGTAAG	ACGTTGGATGCACACAGAACAAGATACGAC	111	92.6	72.3	47.1	44.4	R	5934.9	gGCACAGTAAGGAAAGTGA	T	6206.1	gGCACAGTAAGGAAAGTGAA	C	6222.1	gGCACAGTAAGGAAAGTGAG
rs12337273	ACGTTGGATGAGTGGCTGATACATTGGCTC	ACGTTGGATGACATCTCCACTTGACGTCTC	109	99.7	72.3	47.3	35.0	R	6140.0	AAAGATCAGGAGTTCCATTT	G	6387.2	AAAGATCAGGAGTTCCATTTC	A	6467.1	AAAGATCAGGAGTTCCATTTT
rs2268361	ACGTTGGATGTTGATGCTGTGAGACGAAGG	ACGTTGGATGTTCTTACCAAGAGCTCCCTC	110	99.6	72.3	46.4	50.0	F	6173.0	gtgcGACGAAGGCATCTTGT	C	6420.2	gtgcGACGAAGGCATCTTGTC	T	6500.1	gtgcGACGAAGGCATCTTGTT
rs2059807	ACGTTGGATGATGTGAATCAGACCTCTTGC	ACGTTGGATGAGCCAATAACCATATCAAGG	98	93.0	72.3	48.0	33.3	R	6355.2	AATCAGACCTCTTGCTTTTAA	G	6602.3	AATCAGACCTCTTGCTTTTAAC	A	6682.3	AATCAGACCTCTTGCTTTTAAT
rs2272046	ACGTTGGATGGGATTCAGTAATTGGCCTTG	ACGTTGGATGACATTCTGCATGCATTGTCC	109	96.8	72.3	50.4	52.9	F	6533.2	ggagTGGCCTTGGGACATTTG	C	6780.4	ggagTGGCCTTGGGACATTTGC	A	6804.4	ggagTGGCCTTGGGACATTTGA
rs11692782	ACGTTGGATGACAGTTTCTCAGATCCCTTG	ACGTTGGATGTGGTGTTGTACTTCAGTACG	97	97.1	72.3	50.1	40.9	R	6642.3	TTCTCAGATCCCTTGGTTATTC	T	6913.5	TTCTCAGATCCCTTGGTTATTCA	A	6969.4	TTCTCAGATCCCTTGGTTATTCT
rs12478601	ACGTTGGATGAGAGCTGGAAGTAAAGCCCG	ACGTTGGATGTTCTTTCATTCCTGCTGGTC	93	97.0	72.3	48.4	38.1	R	6740.4	gCGGGTCCTAACATTTTATTGA	T	7011.6	gCGGGTCCTAACATTTTATTGAA	C	7027.6	gCGGGTCCTAACATTTTATTGAG
rs4953616	ACGTTGGATGACTTCATCAGCCACTCTATG	ACGTTGGATGCTACATAACCACACTGAGGG	116	97.6	72.3	47.1	34.8	F	6868.5	CCTCATCATCATTTCCATTATAC	C	7115.7	CCTCATCATCATTTCCATTATACC	T	7195.6	CCTCATCATCATTTCCATTATACT
rs1778890	ACGTTGGATGGAATGTTAAGAATGGTATGG	ACGTTGGATGATGTGGACAGGTAGTGTCAG	116	86.9	72.3	46.1	26.1	F	7058.6	ATTTTCTATAGCAGGTTTATTGA	C	7305.8	ATTTTCTATAGCAGGTTTATTGAC	T	7385.7	ATTTTCTATAGCAGGTTTATTGAT
rs6732721	ACGTTGGATGGACATAGCAGGAGTTGTCAG	ACGTTGGATGTTCCTGTCACTCCATCGTTG	90	99.6	72.3	45.7	40.0	R	7152.7	cggTGTCAGGAAGAGTAATCTAG	T	7423.9	cggTGTCAGGAAGAGTAATCTAGA	C	7439.9	cggTGTCAGGAAGAGTAATCTAGG
rs11891936	ACGTTGGATGCACTCTTAACGTCAATGTCC	ACGTTGGATGGTTCCTATGGTTTCCTTTTC	100	93.0	72.3	45.4	36.8	F	7234.7	tcattTCCTGTTATGCAATTTCTC	C	7481.9	tcattTCCTGTTATGCAATTTCTCC	T	7561.8	tcattTCCTGTTATGCAATTTCTCT
rs2479106	ACGTTGGATGGACTCCTGTCCTTTTGGTTC	ACGTTGGATGACAGGGCACTGGGTTGTTTC	120	97.0	72.3	47.9	36.4	R	7348.8	tgTTGGTTCCTTGATCATAACTAG	G	7596.0	tgTTGGTTCCTTGATCATAACTAGC	A	7675.9	tgTTGGTTCCTTGATCATAACTAGT
rs7857605	ACGTTGGATGAAAGCCCATGAGATCTAGGT	ACGTTGGATGTAGCAACACCTCTGCAAACG	104	97.3	72.3	47.1	30.4	R	7525.9	gaCCTTATTTACTTCTCCAAACATT	T	7797.1	gaCCTTATTTACTTCTCCAAACATTA	C	7813.1	gaCCTTATTTACTTCTCCAAACATTG
rs7371084	ACGTTGGATGCAGTCCCACTATTTAACAGC	ACGTTGGATGCAAGCCTATTATTGGATCCAT	120	85.2	72.3	47.7	38.1	R	7634.0	agacGCAAGTTACTTAACCGATCTA	T	7905.2	agacGCAAGTTACTTAACCGATCTAA	C	7921.2	agacGCAAGTTACTTAACCGATCTAG
rs13429458	ACGTTGGATGATGCACAATGGAGACTGCTG	ACGTTGGATGTAATTAGTGGCAGGGTATAG	99	94.4	72.3	46.9	33.3	F	7738.1	gcttTGCAAAGTTAGAAGATGAAAC	C	7985.3	gcttTGCAAAGTTAGAAGATGAAACC	A	8009.3	gcttTGCAAAGTTAGAAGATGAAACA
rs2292239	ACGTTGGATGGCTATCACCCTTACTTCTGC	ACGTTGGATGACCCTAGATCCCTTAAGTGC	106	99.9	72.3	45.5	33.3	F	7761.1	gggcGTGAAGAGACTTTTGAATCTA	G	8048.3	gggcGTGAAGAGACTTTTGAATCTAG	T	8088.2	gggcGTGAAGAGACTTTTGAATCTAT
rs1894116	ACGTTGGATGAAATTTAGTTGCATTGAGG	ACGTTGGATGAAGGATTGACCACTGTCAAG	113	77.8	72.3	46.7	22.2	R	8231.4	TCTACATAATATTGATTCTAGACAATT	G	8478.6	TCTACATAATATTGATTCTAGACAATTC	A	8558.5	TCTACATAATATTGATTCTAGACAATTT
rs10986105	ACGTTGGATGTCCATCACAATTAGCCTGAG	ACGTTGGATGCACTATAGGCAGTTAAACAA	116	84.5	72.3	50.0	36.4	F	8363.4	gggagTTAGCCTGAGTTATGCAACATA	G	8650.7	gggagTTAGCCTGAGTTATGCAACATAG	T	8690.5	gggagTTAGCCTGAGTTATGCAACATAT
rs4784165	ACGTTGGATGGAGCCAGCCGTACATTAATC	ACGTTGGATGGGAATTTAAGTTATTTTCCC	115	78.6	72.3	49.3	28.6	R	8612.7	GTCACATAATAACTTGAAAAACTATGAG	G	8859.8	GTCACATAATAACTTGAAAAACTATGAGC	T	8883.9	GTCACATAATAACTTGAAAAACTATGAGA

SNP_ID: reference of the sequence; 2nd-PCRP: First Forward Sequence; 1st-PCRP: First Reverse Sequence; AMP_LEN: length of the amplicon; UP_CONF: Uniplex Amplification Score (this score indicates how well the amplicon meets the design criteria, individually); MP_CONF: multiplex amplification score (this score indicates how well the amplicon meets the design criteria, taking into account the other primers included in the multiplex reaction); Tm (NN): melting temperature for the extension primer; PcGC: percentage of GC contained in the first extension; UEP_DIR: Address of the first extension; UEP_MASS: mass of the first extension; UEP_SEQ: sequence of first extension; EXT1_CALL: first allelic variant; EXT1_MASS: mass of the sequence of the first extension + genotype of the first allelic variant; EXT1_SEQ: extension primer sequence + first allelic variant; EXT2_CALL: second allelic variant; EXT2_MASS: mass of the sequence of the first extension + g; EXT2_SEQ: extension primer sequence + second allelic variant.

## Data Availability

Raw data supporting the findings of this study are available upon request from the corresponding authors. Details of the sample included in the study were previously described in the following manuscripts: “Assessment of *THADA* gene polymorphisms in a sample of Colombian women with polycystic ovary syndrome: A pilot study” (DOI: 10.1016/j.heliyon.2022.e09673) and “Study of *LHCGR* gene variants in a sample of Colombian women with polycystic ovarian syndrome: A pilot study” (DOI: doi.org/10.1016/j.jksus.2022.102202).
